# Multiclass Prediction with Partial Least Square Regression for Gene Expression Data: Applications in Breast Cancer Intrinsic Taxonomy

**DOI:** 10.1155/2013/248648

**Published:** 2013-12-30

**Authors:** Chi-Cheng Huang, Shih-Hsin Tu, Ching-Shui Huang, Heng-Hui Lien, Liang-Chuan Lai, Eric Y. Chuang

**Affiliations:** ^1^Graduate Institute of Biomedical Electronics and Bioinformatics, National Taiwan University, No. 1, Section 4, Roosevelt Road, Taipei 10617, Taiwan; ^2^Cathay General Hospital SiJhih, New Taipei, Taiwan; ^3^School of Medicine, Fu-Jen Catholic University, New Taipei, Taiwan; ^4^School of Medicine, Taipei Medical University, Taipei, Taiwan; ^5^Department of Surgery, Cathay General Hospital, Taipei, Taiwan; ^6^Graduate Institute of Physiology, National Taiwan University, Taipei City, Taiwan

## Abstract

Multiclass prediction remains an obstacle for high-throughput data analysis such as microarray gene expression profiles. Despite recent advancements in machine learning and bioinformatics, most classification tools were limited to the applications of binary responses. Our aim was to apply partial least square (PLS) regression for breast cancer intrinsic taxonomy, of which five distinct molecular subtypes were identified. The PAM50 signature genes were used as predictive variables in PLS analysis, and the latent gene component scores were used in binary logistic regression for each molecular subtype. The 139 prototypical arrays for PAM50 development were used as training dataset, and three independent microarray studies with Han Chinese origin were used for independent validation (*n* = 535). The agreement between PAM50 centroid-based single sample prediction (SSP) and PLS-regression was excellent (weighted Kappa: 0.988) within the training samples, but deteriorated substantially in independent samples, which could attribute to much more unclassified samples by PLS-regression. If these unclassified samples were removed, the agreement between PAM50 SSP and PLS-regression improved enormously (weighted Kappa: 0.829 as opposed to 0.541 when unclassified samples were analyzed). Our study ascertained the feasibility of PLS-regression in multi-class prediction, and distinct clinical presentations and prognostic discrepancies were observed across breast cancer molecular subtypes.

## 1. Introduction

Multi-class prediction remains a challenge for high-throughput bioinformatics such as analysis of microarray gene expression data. Numerous machine learning algorithms are readily available for high-throughput data analysis, most of which, however, are limited to scenarios of the classification or prediction with only two classes. This difficulty arises not only from the vast data amount produced by high-throughput microarray or sequencing experiments but from the highly-correlated and nonstochastic nature of genetic/gene expression data. For real-world applications, dichotomous classifications between cancer/normal, alive/dead, and responsive/resistant status are mostly encountered, and many machine learning algorithms and bioinformatics tools perform quite well with sufficient discriminative power [1–3].

One way to tackle the *n* (experimental samples) < *p* (genomic/gene expression features) problem inherited in high-throughput microarray or sequencing techniques is to reduce the high-dimensional data using gene component analysis [4–7]. Gene components, which are synthesized latent factors, and orthogonal transformations of original high-throughput data are interpreted as the projection of high dimensional vector space into a few gene component axes, and the number of gene component (*p*′) is no longer larger than sample numbers (*n*), facilitating the usage of classical statistical tools.

In previous work we demonstrated that gene component analysis could discriminate estrogen receptor (ER) positive and negative breast cancers and gene component classifiers could be projected into independent samples with high predictive accuracy, as well as an integrated step of automatic gene selection [8]. We also concluded that principle component (PC) regression was more suitable for unsupervised class discovery while partial least square (PLS) was more efficient in supervised class prediction.

The aim of the study was to apply PLS-regression for breast cancer intrinsic taxonomy, of which five distinct molecular subtypes were identified from microarray experiments. Here we extended the applications of PLS-regression from two-class (ER positive versus ER negative and Luminal-A versus Luminal-B subtype) into multiclass prediction of the full spectrum of breast cancer intrinsic taxonomy [9]. We hypothesized that PLS-regression could be an alternative and efficient classification algorithm for breast cancer microarray experiments pertaining intrinsic signature genes.

## 2. Materials and Methods

### 2.1. Breast Cancer Intrinsic Taxonomy

In the past decade, microarray experiments have redefined breast cancers as heterogeneous diseases in terms of molecular aberrations, and a number of taxonomic classifications based on gene expression profiles that have been reported have shown some prognostic significance. One such molecular taxonomy is the “intrinsic subtype” proposed by the Stanford/UNC group. Perou identified 476 intrinsic genes from 65 patients with breast cancers and normal individuals; four subclasses: basal-like, Erb-B2+, normal breast-like, and luminal epithelial/ER+ were revealed by class discovery through clustering analysis [10, 11]. The luminal subtype was further divided into luminal-A and luminal-B, and distant metastases were strongly associated with the expression patterns of intrinsic genes [12]. Independent studies supporting the existence of breast cancer intrinsic subtypes followed [13, 14]. By definition, intrinsic genes were those genes that show the highest variation across different subjects and show the least variation within each individual (i.e., pre-/post-chemotherapy changes) [12]. The latest version of intrinsic signature, prediction analysis of microarray 50 gene set (PAM50), was supposed to provide prognostic and predictive values independent of traditional prognostic factors such as hormone receptor, human epidermal growth factor receptor 2 (HER2) status, or proliferation markers [15].

The PAM50 intrinsic signature genes that defined 5 molecular subtypes (luminal-A, luminal-B, normal breast-like, HER2-enriched, and basal-like) were retrieved. The expression values of training samples deriving intrinsic signatures were downloaded from UNC Microarray Database (https://genome.unc.edu/). Centroids were the mean expression values of intrinsic genes corresponding to each molecular subtype. The prototypes included 12 normal breast-like, 57 basal-like, 35 HER2-enriched, 23 luminal-A, and 12 luminal-B tumors.

### 2.2. PLS-Regression Classifier

Following identification of intrinsic genes, PLS was used for dimension reduction and latent X-factors (gene components) construction. The troublesome *n* (sample size) < *p* (gene expression predictors) problem became tractable since a much smaller *p*′ (gene component) was used instead of original microarray gene expression features. At the same time model over-fitting and collinearity of original *p* genes was avoided due to the limited number of gene components (*p*′) used in classification algorithm and the uncorrelated nature between successive latent factors.

All gene component regressions were essentially the linear transformations of original gene expression values and could be viewed as the projection of high dimensional predictor space into a few orthogonal latent factor axes. PLS maximized the covariance between the predictor and response variables. In matrix algebra, let *X*_0_ and *Y*_0_ be centered and scaled matrix of predictive and responsive variables; one dummy variable *Y* indicating clinical phenotype was needed for binary classifications in PLS. PLS maximized *X*_0_′*Y*_0_ for latent factor construction. PLS predicted *X*_0_ (and *Y*_0_) with the following formula:

(1) X0=tp′, where  p′=(t′t)−1t′X0,Y0=uc′, where  c′=(u′u)−1u′Y0.

The *x*-scores (*t* = *X*_0_ *w*) and *y*-scores (*u* = *Y*_0_ *q*) were derived to meet the criteria of maximal covariance of *t*′*u* where *w* and *q* were associated weighted vectors. The vectors *p* and *c* were *x*- and *y*-loadings, respectively. It should be noticed that all latent factor extractions were under orthogonal constraints; successive latent factors (gene components) were linearly independent to each other, and usually the corresponding eigen-vectors were normalized to unity (*standardized* linear combinations of original variables or *orthonormal *transformations).

The number of latent factors used for PLS-regression was determined by cross-validation. We used split-sample cross validation to determine the number of latent factors that delivered the minimal predicted residual sum of squares (PRESS) followed by van der Voet's test; the fewest number of gene components that was insignificantly different from the factor number corresponding to the minimal PRESS should be used in regression [16]. In short, it was a randomization-based model comparison test performed on each cross-validation model [17]. Missing values in gene expression values were handled by imputing the missing ones with the non-missing values for the corresponding variable first, then followed by filling in missing values with their predicted values based on that fitted model and computed the model again (expectation-maximization algorithm).

After the number of gene component was determined and each gene component score was calculated for all samples, binary logistic regression (LR) was applied for classification/prediction. For binary LR, the predicted probability was estimated by

(2) (1+exp⁡−(β0+β1×1+⋯+βk×k))−1,

where *k* was the number of gene component used, and *x*_*k*_ was the *k*th gene component score. To evaluate classifier performance, leave-one-out cross validation was used to prevent model over-fitting. The threshold of a positive prediction was defined to have a more than 0.5 of cross-validated predicted probability. The process of PLS scores construction and LR prediction was repeated for each of the 5 molecular subtypes. Bonferroni corrections with a reduced *α* level of 0.01 were applied for all PLS-regression classifiers for multiple comparisons. All samples were categorized into one of the 5 molecular subtypes with the highest predicted probability, assuming that probability exceeding the threshold of 0.5. An ambiguous classification was claimed when more than one predicted probability was higher than 0.5 among all subtypes. A sample was designated as unclassifiable if none of the predicted probabilities of 5 molecular subtypes exceeded the threshold of 0.5.

In each classifier, a binary PLS-regression was fit, with the most relevant genes associated with the subtype enrolled as predictive variables. Each classifier compromised 10 out of the 50 PAM50 signature genes, and this class-specific gene selection avoided using all 50 genes into the PLS regression at the same time.

### 2.3. Validation Dataset

Our microarray experiments and two publicly available microarray studies fulfilled the purpose of external validation [18–20]. Our study material included 83 breast cancers from Taiwan (GSE48391); sporadic breast cancer samples were collected consecutively during surgery, snapped frozen in liquid nitrogen, and then stored at −80°C. The frozen samples were dissected into slices of 1-2 mm thickness, and more than 90% of cancerous content was a pre-requisite for microarray experiments. Written consent was obtained for all subjects before sample collection with the protocol approved by Institute Review Board of Cathay General Hospital. The criteria of enrolment included incident/invasive breast cancers without neo-adjuvant therapy, no systemic spread (clinical stage I to III), no concurrent secondary malignancy, and less than 70 years of age. Enrolled patients were managed according to standard guidelines with regular follow-up.

For relevant pathological features, ER positivity was defined as the presence of at least 10% of nuclei with positive results by immunohistochemical (IHC) analysis, and breast samples displaying low ER positivity (<10% of nuclei with positive stains) were not assayed in the current study. For HER2 status, the ASCO and CAP guidelines were followed: IHC3+ and IHC2+ with fluorescence in situ (FISH) hybridization amplification were considered to indicate HER2 overexpression.

Total RNA from cancerous breast tissues was extracted by TRIzol reagent (Invitrogen, Carlsbad, CA) and RNA was purified using RNeasy mini kits (Qiagen, Germantown, MD). RNA integration was tested by gel electrophoresis. Affymetrix (Affymetrix, Santa Clara, CA) GeneChip Human Genome U133 plus 2.0 was used for the microarray experiment. Hybridization and scanning were performed according to the Affymetrix standard protocol. Images were scanned using GeneChip Scanner 3000, and the scanned images were processed with GeneChip Operating Software (GCOS). Robust multi-array average (RMA) algorithm was used to normalize 83 array chips [21].

Two publicly available breast cancer microarray depositories, one from Lu et al. and another from Kao et al., were merged with our microarrays to form the validation dataset [18–20]. Both datasets used the same Affymetrix U133 plus 2.0 microarrays as used in our experiments, and all assayed subjects were Han Chinese ethnically. RMA was used for normalization within each dataset [21]. Details of microarray experiments and the demography of the study populations had been described elsewhere [19, 20]. The Lu et al. dataset comprised 125 Chinese breast cancers with known clinical ER and HER2 status, and original Affymetrix CEL files were downloaded from NCBI Gene Expression Omnibus (GSE5460); clinical ER and HER2 status was provided. For the Kao et al. dataset, 327 Taiwanese breast cancers were assayed, and corresponding disease-free survival and overall survival data were available (GSE 20685). The median follow-up time of our 83 breast cancer patients was 3.7 years (range: 0.1 to 5.8 years) with 13 events of recurrence, metastasis, or breast cancer-specific mortality (16%) and 11 deaths (all-cause mortality). For 327 breast cancers from Kao et al. (GSE20685), the median follow-up was 7.7 years with 94 events of recurrence, metastasis, or breast cancer-specific mortality (29%), and 83 deaths (all-cause mortality).

All intrinsic genes were mapped to the Affymetrix gene annotation file, and only the most variable probeset measured by inter-quartile range (IQR) across all arrays was used when multiple probesets per gene were encountered. The 535 breast cancer specimens of the Han Chinese patients were assigned to 1 of the 5 molecular subtypes with the nearest centroid method (single sample prediction, SSP). Spearman's rank correlation coefficients were used, and samples were designated as unclassified if correlation coefficients to all 5 centroids were less than 0.1. To enhance the comparability between the original studies deriving intrinsic genes and independent samples in current study, mean-centering of genes was applied to the expression data of Han Chinese breast cancers, as suggested by the investigators of the Stanford group [22]. All arrays within each study were scaled and centered (mean = 0 and standard deviation = 1) on a gene-by-gene basis before PLS-regression was performed in order to overcome the discrepancies and enhance comparability across microarray studies.

## 3. Results

### 3.1. PLS-Regression in Prototypical Arrays

PLS-regression classifiers based on latent gene component scores were built for each molecular subtype from training dataset of 139 prototypical arrays.  Table 1 showed the performance of individual classifiers. The number of gene component chosen for PLS regression ranged from 1 to 2.  Table 2 tabulated PAM50 prototypes with class labels predicted by PLS- regression. The agreement between PAM50 prototypes and predicted subtype by PLS-regression was excellent (weighted Kappa: 0.988, 95% CI: 0.965–1) after excluding 16 unclassified cases. It should be noted that six cases were ambiguously predicted into luminal-A (*n* = 4), luminal-B, and normal breast-like subtype since these cases were positively predicted by two classifiers.

### 3.2. PLS-Regression in Validation Arrays

PLS-regression was performed for independent Han Chinese breast cancers including our series and two publicly available microarray depositories. To derive the “gold standard” for intrinsic subtype, centroid-based method (SSP) was used to designate each individual of the three studies into 1 of the 5 molecular subtypes.

Since no missing value was found in Affymetrix microarrays used for validation, there was no need of missing value imputations.  Table 3 showed the results of PLS-regression classifiers with centroid-based SSP as the gold standard. At most two gene components were adopted by PLS-regression.  Table 4 compared the results of PLS-regression and subtypes designated by SSP. A much compromised agreement between SSP and PLS-regression was observed, with only a fair weighted Kappa statistic of 0.541 (95% CI: 0.486–0.597) reported. The number of ambiguous cases raised to 55. Around one-fourth (*n* = 125) of tested samples were categorized as unclassified by PLS-regression.

### 3.3. Clinical Presentations and Prognostic Discrepancies among Intrinsic Taxonomy

Clinical and follow up data were available for 208 of Han Chinese breast cancers and we compared ER and HER2 phenotypes between distinct intrinsic subtypes designated by PAM50 SSP and PLS-regression (Table 5). Despite fewer cases analyzed by PLS-regression due to more unclassified samples, characteristics of molecular subtypes were similar between predictive results of PAM50 SSP and PLS-regression.

**Figure 1:**
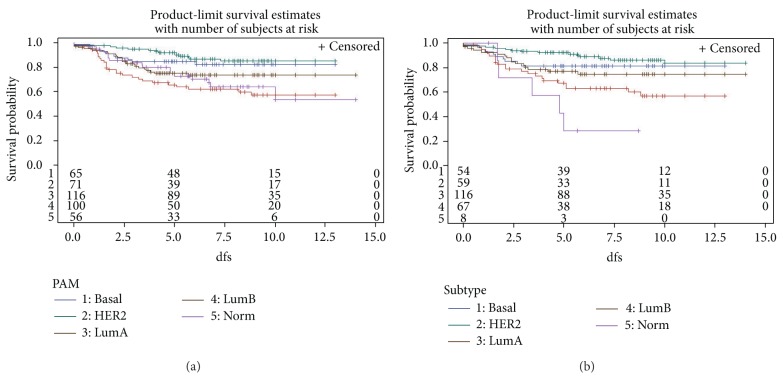
Breast cancer disease-free survival stratified by intrinsic subtypes, classified by either PAM50 single sample prediction (a) or PLS-regression (b). dfs, disease-free survival; Basal, basal-like; HER2, Her2-enriched; LumA, luminal-A; LumB, luminal-B; Norm, normal breast-like subtype.

Figures 1(a) and 1(b) showed disease-free survival of 410 Han Chinese breast cancers with follow up data, classified by PAM50 SSP and PLS-regression, respectively. As expected, luminal-A subtype reported more optimistic results of breast cancer therapy. The prognoses of molecular subtypes other than luminal-A were much more intertwined and compromised.

## 4. Discussion 

In the current study, PLS-regression was used for microarray multiclass predictions. Latent gene component scores were used in binary LR, each time with one molecular subtype tested. For breast cancer intrinsic taxonomy, PLS-regression classifiers were built for five mutually exclusive molecular subtypes. Bonferroni corrections were applied for multiple comparisons (5 times of classifications per each case). If the cross-validated predicted probability was higher than 0.5, a positive prediction was recognized. For most instances, there was only one classifier reported a positive prediction and the sample was categorized into the corresponding subtype. If two classifiers reported a higher than 0.5 predicted probability, the case was classified into the subtype with the highest probability but an ambiguous prediction was identified. If all classifiers failed to deliver a prediction higher than the threshold of 0.5 cross-validated probability, an unclassified sample was claimed.

Applications of gene component methodology for microarray studies had been reported in literature. West et al. demonstrated the “metagene” model, which used principle component (PC) scores from the top 100 genes showing the highest absolute correlations with clinical ER status of breast cancers and used these PC scores as predictive variables in binary regression [23]. Following studies adopting “metagene” concept, which was PC approach in nature, utilized the gene component scores in Bayesian classification tree [24]. On the other hand, Nguyen and Rocke performed binary and polychotomous LR and linear/quadratic discriminative analysis from PLS scores for two-class and multi-class microarray tumor classification problems [5, 6]. The main difference between PC and PLS is that PC extracts latent factors accounting for most of gene expression variations regardless of outcome variables and is unsupervised while PLS maximizes the covariance between latent explanatory and latent dependent variables and is supervised in nature. For this reason, it was postulated that PLS might perform better than PC in microarray classification problem and indeed, successful results of microarray gene component classification with PLS had been reported for several human cancers in past few years [25, 26]. Our previous studies compared predictive performance of gene component approaches, and concluded that PC regression was more suitable for unsupervised class discovery while PLS was more efficient in supervised class prediction [8].

Since PLS automatically produced (predicted) response variable (tumor class label), one-step PLS regression, which predicted tumor class directly from latent *y*-scores was reported by Pérez-Enciso and Tenenhaus [7]. However, for breast cancer intrinsic taxonomy comprising five molecular subtypes, at least four dummy variables were required, and the mutual exclusive relationships between these responsive variables were not constrained. For these reasons, direct PLS modeling of five molecular subtypes was not practical.

Multi-class prediction of PLS-regression was the extension of the regression for binary responses. The strategy of latent score construction remained the same. It was quite intuitive that polychotomous (ordinal or nominal) LR could fill the task of prediction with multiple responsive levels. However, neither ordinal (with one baseline class) nor nominal LR resulted in a converged model in the training or validation dataset. Multi-class prediction remained a challenge for high-throughput gene expression data analysis with classical statistical tools.

**Figure 2:**
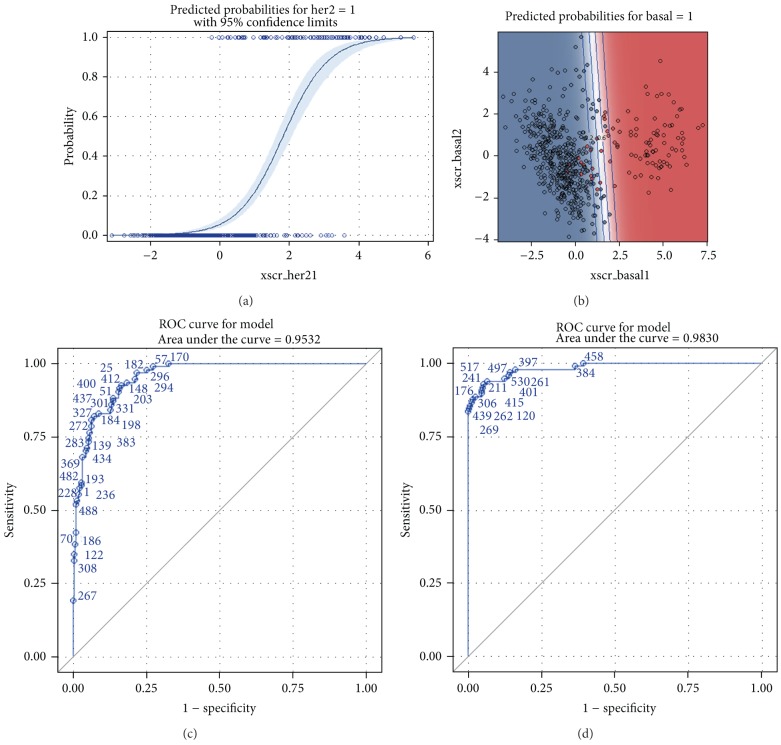
Predicted probabilities and 95% confidence interval as a function of the 1st PLS score or 1st/2nd PLS scores for HER2-enriched and basal-like subtype (a and b) and corresponding ROC curves (c and d). (*x*scr_her21: the 1st *x*-score for HER2-enriched subtype, *x*scr_basal1: the 1st *x*-score for basal-like subtype, *x*scr_basal2: the 2nd *x*-score for basal-like subtype).

To overcome aforementioned difficulties, our strategy started with the development of PLS-regression for each of the molecular subtypes individually. In each classifier a binary PLS-regression was fit, with the most relevant genes associated with the subtype enrolled as predictive variables.  Table 6 showed the compositions and weight vectors of PLS regressions for each intrinsic subtype. It deserved notice that each classifier compromised 10 out of the 50 PAM50 signature genes, and this class-specific gene selection avoided using all 50 genes into the PLS regression at the same time. These class-specific predictors for PLS-regression were not a coincidence but were revealed in tFhe intermediate step when PAM50 signature genes were selected. It was the ClaNC (classification to nearest centroids) algorithm which determined the composition of these class-specific genes [27]. More details could be disclosed from Figure 2(a) from the original publication of PAM50 [15].

In 139 prototypical arrays, the agreement between PAM50 SSP and PLS-regression was excellent (weighted Kappa: 0.988), indicating the robustness and feasibility of PLS-regression as an alternative classification method to PAM50 SSP. In validation dataset of 535 Han Chinese breast cancer microarrays, the agreement between PAM50 SSP and PLS-regression deteriorated substantially. If we took a close look at Table 4, the compromised performance of PLS-regression in independent samples largely resulted from increased number of unclassified cases. If these unclassified samples were removed, the agreement between PAM50 SSP and PLS-regression improved enormously (weighted Kappa: 0.829 as opposed to 0.541 when unclassified samples were analyzed). Another clue came from the fact that if we forced all samples to be categorized into the subtype with the highest predicted probability (given that the highest probability was more than 0.1 as was in PAM50 SSP), the agreement between PAM50 SSP and PLS-regression was ameliorated with a weighted Kappa of 0.704 (95% CI: 0.649–0.758). The unclassified samples reduced to 9 (2% of 535 assayed samples).

An apparent benefit of PLS-regression rather than centroid-based SSP proposed by PAM50 investigators was that the predictive probability was reported. In our study we used the 0.5 of (cross-validated) predicted probability as the threshold of a positive prediction. If the threshold was relaxed to a lower level, the number of unclassified cases decreased but was with the expense of increased ambiguous classifications (two or more than two classifiers reported a positive prediction). Although we could designate samples into the class with the highest predictive probability, there remained a doubt about the validity of molecular taxonomy when more than one classifier passed the predefined threshold and reported a positive prediction. With current threshold of 0.5, there were fewer than 10% of cases with ambiguous classifications.

In centroid-based SSP, since samples were categorized into the subtype with the highest correlation coefficient, and the unclassified threshold was set to a much lower level (less than 0.1 of correlation coefficients to all five centroids), the higher proportion of unclassified cases of PLS-regression in independent dataset was not a drawback of purposed algorithm but indicated a more precise and sophisticated statistical rationale. In our opinion, the threshold of 0.1 correlation coefficient in PAM50 SSP was too loose as the proportion of unclassified cases was erroneously reduced with the expense of compromised reproducibility and robustness. The threshold of positive predicted probability could be viewed as a tuning parameter of PLS-regression, as suggested by high area under the curve (AUC) values of most classifiers (Tables 1 and 3). In the current study, the threshold of 0.5 implied an uninformative prior and an unprejudiced belief in individual classifier of each molecular subtype. Figures 2(a) and 2(b) showed the predicted probability as a function of the latent PLS scores, with one and two gene components incorporated into regression for HER2-enriched and basal-like subtype classifier, respectively. Figures 2(c) and 2(d) showed the corresponding receiver operating characteristic (ROC) curves.

The clinical ER and HER2 status for 208 Han Chinese breast cancers were presented in Table 5; no basal-like breast tumors were ER positive, and most HER2-enriched breast tumors (around four-fifths) were clinically ER negative, whereas most of luminal-A and luminal-B subtypes were ER positive. For clinical HER2 status, most basal-like breast tumors were HER2-normal, most HER2-enriched subtype cases were with HER2 over-expression, and the luminal-B tended to report a higher propensity of HER2 over-expression than the luminal-A subtype, regardless of predictive methods (PLS-regression or PAM50 SSP). Thesse findings, in general, were in agreement of what we learnt from previous studies about intrinsic taxonomy and further evidenced the validity of current study [20]. Disease-free survival from 410 cases of validation dataset was displayed in Figures 1(a) and 1(b). The luminal-A subtype was associated with the best prognosis during the follow-up for Han Chinese breast cancers.

Tables 3 and 4 showed that the agreement between PAM50 SSP and PLS-regression for normal breast-like subtype was extremely low, also indicated by the unsatisfactory adjust *R*-square as well as compromised sensitivity. Notably, normal breast-like centroid in PAM50 was derived from 29 normal breast samples; in 2009 Parker et al. clained that normal breast-like category of PAM50 should be treated as an internal quality control rather than a breast cancer intrinsic subtype such as normal breast-like subtype in Hu 306 and Sørlie 500 intrinsic signatures [15]. In this sense, none of our samples should be predicted as normal breast-like subtype with PAM50. In our study, the number of samples categorized as the normal breast-like subtype by PLS-regression (*n* = 8) was less than the number designated as normal breast-like by PAM50 SSP (*n* = 56), indicating a more precious and valid prediction of the purposed gene component algorithm. The dubious clinical meaning and doubtful existence of normal breast-like subtype, also reflected in its heterogeneous clinical presentations of ER and HER2 phenotypes, remained unsolved and demanded further evaluations. Perhaps directly assaying true normal breast tissues might shed light on this issue.

## 5. Conclusion

Our study extended the applications of PLS-regression for gene expression data to multi-class taxonomy such as PAM50 intrinsic subtypes purposed by the Stanford/UNC group. With gene component classifiers and class-specific genes for each molecular subtype, the purposed algorithm was validated in original cohort deriving the PAM50 signature as well as in independent Han Chinese breast cancers with modest sample size. PLS-regression was evidenced to be a feasible and efficient alternative to centroid-based SSP when more than two classes were discerned. The increased proportion of unclassifiable cases in independent samples deserved meticulous evaluation. Whether inconsistency in classification threshold or unrecognized patterns in full spectrum of intrinsic taxonomy resulted in these undetermined cases was speculated; further gene expression studies might be directed to answer these questions in an effort to derive a more sophisticated signature for human breasts cancer.

## Figures and Tables

**Table 1:** Performance of PLS-regression classifiers for prototypical arrays.

Intrinsic subtype	Basal-like	HER2-enriched	Luminal-A	Luminal-B	Normal breast-like

Number of samples	57	35	23	12	12
PLS-regression					
Number of gene component	1	1	2	1	2
*X*-variance explained	57.0%	37.1%	74.5%	25.8%	60.2%
*Y*-variance explained	86.7%	56.2%	64.6%	24.6%	66.5%
Binary LR					
Adjusted *R*-square	0.99	0.73	0.9	0.63	0.99
AUC	1	0.96	0.99	0.96	1
Accuracy	98.6%	89.9%	97.1%	95.0%	100.0%
Sensitivity	98.2%	74.3%	91.3%	50.0%	100.0%
Specificity	98.8%	95.2%	98.3%	99.2%	100.0%

PLS: partial least square, LR: logistic regression, AUC: area under the curve.

**Table 2:** PAM50 prototypes and predicted subtypes by PLS-regression for prototypical arrays.

PAM50 prototype (sample number)	Predicted subtype					
	Basal-like	HER2-enriched	Luminal-A	Luminal-B	Normal breast-like	Unclassified
Basal-like (57)	57	0	0	0	0	0
HER2-enriched (35)	0	26	0	0	0	9
Luminal-A (23)	0	0	21	0	0	2
Luminal-B (12)	0	1	0	6	0	5
Normal breast-like (12)	0	0	0	0	12	0

**Table 3:** Performance of PLS-regression classifiers for independent validation dataset.

Intrinsic subtype	Basal-like	HER2-enriched	Luminal-A	Luminal-B	Normal breast-like

Number of samples	97	94	165	121	56
PLS-regression					
Number of gene component	2	1	2	2	1
*X*-variance explained	71.1%	25.5%	79.9%	61.7%	38.1%
*Y*-variance explained	56.9%	41.6%	34.5%	30.9%	18.1%
Binary LR					
Adjusted *R*-square	0.86	0.66	0.73	0.61	0.39
AUC	0.98	0.95	0.95	0.93	0.89
Accuracy	96.6%	90.7%	88.2%	86.2%	90.5%
Sensitivity	85.6%	68.1%	81.8%	63.6%	23.2%
Specificity	99.1%	95.5%	91.1%	92.8%	98.3%

PLS: partial least square, LR: logistic regression, AUC: area under the curve.

**Table 4:** Single sample prediction by PAM50 centroids and predicted subtypes by PLS-regression for independent validation dataset.

PAM50 SSP (sample number)	Predicted subtype					
	Basal-like	HER2-enriched	Luminal-A	Luminal-B	Normal breast-like	Unclassified
Basal-like (97)	83	1	0	1	0	12
HER2-enriched (94)	0	63	0	3	0	28
Luminal-A (165)	0	3	130	8	0	24
Luminal-B (121)	0	5	10	73	0	33
Normal breast-like (56)	1	3	17	1	8	26
Unclassified (2)	0	0	0	0	0	2

**Table 5:** Association of clinical ER and HER2 status with intrinsic taxonomy, classified by either PAM50 single sample prediction or PLS-regression.

	Basal	HER2	LumA	LumB	Norm
ER	PAM50 SSP				
Negative	40	28	0	3	9
Positive	0	7	67	46	7
HER2					
Normal	37	5	63	30	8
Over-expression	3	30	4	19	8

ER	PLS-regression				
Negative	38	19	1	1	3
Positive	0	5	59	33	2
HER2					
Normal	35	0	56	21	4
Over-expression	3	24	4	13	1

SSP: single sample prediction, Basal: basal-like, HER2: Her2-enriched, LumA: luminal-A, LumB: luminal-B, Norm: normal breast-like subtype.

**Table 6:** Compositions and weight vectors of five PLS-regressions for each molecular subtype.

Basal-like		HER2-enriched		Luminal-A		Luminal-B		Normal breast-like	
ANLN	0.271	ACTR3B	−0.316	BIRC5	−0.299	BCL2	−0.325	CCNB1	−0.272
CEP55	0.271	BAG1	−0.083	CDCA1	−0.294	CDH3	−0.667	CDC6	−0.241
ESR1	−0.319	BLVRA	0.317	CENPF	−0.288	CXXC5	0.484	KRT14	0.350
FOXA1	−0.417	CCNE1	−0.067	EXO1	−0.293	EGFR	−0.316	KRT17	0.241
FOXC1	0.370	CDC20	−0.069	MAPT	0.352	KIF2C	−0.050	KRT5	0.276
GPR160	−0.297	ERBB2	0.452	MYBL2	−0.328	MDM2	0.027	MLPH	0.376
KNTC2	0.303	FGFR4	0.365	NAT1	0.421	MKI67	−0.136	MMP11	−0.404
MELK	0.270	GRB7	0.470	PTTG1	−0.299	ORC6L	−0.049	RRM2	−0.359
MIA	0.296	MYC	−0.390	SLC39A6	0.339	PR	−0.143	TYMS	−0.286
TMEM45B	−0.323	SFRP1	−0.343	UBE2C	−0.296	PHGDH	−0.529	UBE2T	−0.374
